# Berberine Improves Chemo-Sensitivity to Cisplatin by Enhancing Cell Apoptosis and Repressing PI3K/AKT/mTOR Signaling Pathway in Gastric Cancer

**DOI:** 10.3389/fphar.2020.616251

**Published:** 2020-12-09

**Authors:** Yingying Kou, Bending Tong, Weiqing Wu, Xiangqing Liao, Min Zhao

**Affiliations:** ^1^ GCP Office, Jiangsu Cancer Hospital, Jiangsu Institute of Cancer Research, The Affiliated Hospital of Nanjing Medical University, Nanjing, China; ^2^ Department of Pharmacy, Jiangsu Cancer Hospital, Jiangsu Institute of Cancer Research, The Affiliated Hospital of Nanjing Medical University, Nanjing, China; ^3^ Department of Health Management, First Affiliated Hospital of Southern University of Science and Technology, Second Clinical College of Jinan University, Shenzhen, China

**Keywords:** gastric cancer, cisplatin, berberine, apoptosis, PI3K/AKT/mTOR 2

## Abstract

Gastric cancer is one of the most common malignancies ranks as the second leading cause of cancer-related mortality in the world. Cisplatin (DDP) is commonly used for gastric cancer treatment, whereas recurrence and metastasis are common because of intrinsic and acquired DDP-resistance. The aim of this study is to examine the effects of berberine on the DDP-resistance in gastric cancer and explore the underling mechanisms. In this study, we established the DDP-resistant gastric cancer cells, where the IC_50_ values of DDP in the BGC-823/DDP and SGC-7901/DDP were significantly higher than that in the corresponding parental cells. Berberine could concentration-dependently inhibited the cell viability of BGC-823 and SGC-7901 cells; while the inhibitory effects of berberine on the cell viability were largely attenuated in the DDP-resistant cells. Berberine pre-treatment significantly sensitized BGC-823/DDP and SGC-7901/DDP cells to DDP. Furthermore, berberine treatment concentration-dependently down-regulated the multidrug resistance-associated protein 1 and multi-drug resistance-1 protein levels in the BGC-823/DDP and SGC7901/DDP cells. Interestingly, the cell apoptosis of BGC-823/DDP and SGC-7901/DDP cells was significantly enhanced by co-treatment with berberine and DDP. The results from animals also showed that berberine treatment sensitized SGC-7901/DDP cells to DDP *in vivo*. Mechanistically, berberine significantly suppressed the PI3K/AKT/mTOR in the BGC-823/DDP and SGC-7901/DDP cells treated with DDP. In conclusion, we observed that berberine sensitizes gastric cancer cells to DDP. Further mechanistic findings suggested that berberine-mediated DDP-sensitivity may be associated with reduced expression of drug transporters (multi-drug resistance-1 and multidrug resistance-associated protein 1), enhanced apoptosis and repressed PI3K/AKT/mTOR signaling.

## Introduction

Gastric cancer is one of the most common malignancies ranks as the second leading cause of cancer-related mortality in the world (Thrift and El-Serag, 2020). Around 30% of the total cases were diagnosed in China (Leung et al., 2008; Piñeros et al., 2017). Recently, great advancements have been made in early diagnosis and therapeutic treatments including surgical resection and chemo-/radio-therapy, whereas the clinical outcomes of the patients with this malignancy remains poor (Van Cutsem et al., 2016). Cisplatin (DDP) as a chemotherapeutic reagent has been widely used in the treatment for gastric cancer, and DDP-based therapy could significantly improve the survival of patients with gastric cancer (Wagner et al., 2017). Unfortunately, metastasis and recurrence of the gastric cancer are commonly existing in the patients due to the acquired and intrinsic DDP resistance (Haller and Misset, 2002; Baek et al., 2012; Choi et al., 2017). As DDP is still the standard chemotherapy for gastric cancer, developing effective way to reduce the DDP-resistance in gastric cancer is of great clinical significance.

The compounds from the herbal medicine have been attracting attentions due to their anti-cancer activities (Wang et al., 2018). Berberine is an iso-quinoline alkaloid and can be extracted from Beberis species (Imenshahidi and Hosseinzadeh, 2016; Wang et al., 2017). Studies have demonstrated that berberine possessed various pharmacological actions including anti-hypertensive, anti-arrhythmic, anti-bacterial and anti-cancer effects (Imenshahidi and Hosseinzadeh, 2016; Wang et al., 2017). Furthermore, studies found that berberine attenuated the radio-resistance of colon cancer cells via repressing P-gp expression (Guan et al., 2020). Gao et al., found that berberine could sensitize breast cancer cells to different chemotherapeutic drugs (Gao et al., 2019). Liu et al., showed that berberine could attenuate the DDP-resistance of ovarian cancer cells by targeting miR-21/programmed cell death 4 axis (Liu et al., 2013). Pre-treatment with berberine was effective to promote the anti-tumor effects of DDP in laryngeal cancer cells (Palmieri et al., 2018). Studies from Pandey et al., showed the potential actions of berberine to attenuate 5-fluoruracil-resistance in gastric cancer cells (Pandey et al., 2015); however, the exact actions of berberine in the DDP-resistance are not fully explored.

In the present study, we firstly established the DDP-resistant cellular model using two gastric cancer cell lines (BGC-823 and SGC-7901) under elevated concentrations of DDP. After that, we explored if berberine could attenuate the drug-resistance in these cell lines and deciphered the potential molecular mechanisms. This study may provide a novel strategy for managing the DDP-resistance in gastric cancer.

## Materials and Methods

### Cell Lines and Generation of DDP-Resistant Cells

The BGC-823 and SGC-7901 cells were purchased from the Shanghai Institutes for Biological Sciences, Chinese Academy of Sciences (Shanghai, China) and the cells were cultured according to the instructions. For the generation of DDP-resistant BGC-823 (BGC-823/DDP) and DDP-resistant SGC-7901 (SGC-7901/DDP) cells, the parental cells (BGC-823 and SGC-7901) were initially treated with 0.5 µM DDP; and then the concentrations of DDP were gradually increased to 1, 3 and 10 µM (the highest concentration) every days. BGC-823 and SGC-7901 cells became resistant to DDP (10 µM) were chosen for further experimentation.

### Drug Treatments

The chemicals including DDP (catalogue #1134357) and berberine (catalogue #200275; purity ≥95%) were purchased from Sigma-Aldrich (St. Louis, United States). For the DDP treatments, the BGC-823, SGC-7901, BGC-823/DDP and SGC-7901/DDP cells were treated with increased concentrations of DDP (1, 3, 10, 30, 100 and 300 µM) for 24 h; for the berberine treatments, these cells were treated with increased concentrations of berberine (1, 3, 10, 30, 100, 300 and 1,000 µM) for 24 h; for the co-treatments, these cells were co-treated with DDP and berberine at different concentrations for 24 h. After the drug treatments, these cells were harvested for further *in vitro* analysis.

### 3-(4,5-Dimethylthiazol-2-yl)-2,5-Diphenyltetrazolium Bromide (MTT) Assay

The effects of DDP and berberine on the cell viability were determined by (3-(4,5-dimethylthiazol-2-yl)-2,5-diphenyltetrazolium bromide) MTT assay. Different cell lines with respective treatments were seeded in triplicate in a 96-well plate, and after incubating at 37°C for 24 h, the cells were incubated with 5 mg/ml MTT reagent in phosphate buffered saline at 37 °C for 2 h. After that, the 50% dimethyl formamide was added to solubilize the formazan crystals. Finally, the optical density (OD) values at 570 nm wavelength was determined using the microplate reader. Cell viability (%) was calculated as follows: (OD in the experimental group/OD in the control group) * 100. The IC_50_ values were analyzed using the non-linear regression fit.

### Caspase-3 and Capsase-9 Activities Determination

Caspase-3 and caspase-9 activities of BGC-823/DDP and SGC-7901/DDP cells with respective treatments were assessed using the commercial caspase-3 and -9 activity kits, respectively (Beyotime, Beijing, China) according to the supplier’s protocols.

### Flow Cytometry for Cell Apoptosis

Cell apoptosis of BGC-823/DDP and SGC-7901/DDP cells were assessed using the propidium iodide (PI) and fluorescein isothiocyanate (FITC)-Annexin V Apoptosis Detection kit (Thermo Fisher Scientific). BGC-823/DDP and SGC-7901/DDP cells with respective treatments were harvested and stained with PI and FITC-Annexin V according to the supplier’s protocols. The percentage of Annexin V-positive population cells was assessed using a Calibur Flow cytometer (BD Biosciences, Franklin Lake, United States), and the cell apoptotic rates were determined using Flow Jo software (Version 7.6.1, TreesStar, Ashland, United States).

### Western Blot Analysis

BGC-823/DDP and SGC-7901/DDP cells with respective treatments were lysed with Radioimmunoprecipitation assay buffer supplied with the protease inhibitors cocktail (Sigma, St. Louis, United States) on ice for at least 15 min. The protein samples were collected by obtaining the supernatants after centrifugation (12,000 *g*) for 20 min at 4°C. The concentration of the protein samples was determined using the bicinchoninic acid protein assay kit (Beyotime) according to the supplier’s protocol. A total of 45 µg proteins were separated on the sodium dodecyl sulphate-polyacrylamide gel electrophoresis followed by transferring to the polyvinylidene difluoride membranes (Millipore) by using the electrophoretic method. After blocking with non-fat milk (5%) in Tris-buffered saline with 0.1% Tween-20 (TBST), the membrane was washed with TBST followed by incubating with corresponding primary antibodies at 4°C for 16 h. After that, the membrane was washed with TBST for 3 × 5 min followed by incubating with the membrane was then washed with TBST three times, followed by incubation with a horseradish peroxidase-conjugated secondary antibody (Cell Signaling Technology) at room temperature for 2 h. The blot bands on the membrane was detected using the enhanced chemiluminescence kit (Thermo Fisher Scientific) according to the supplier’s protocol. The protein expression levels were evaluated using densitometric method using the Image J software. The concentrations and the sources of the primary antibodies were shown below: β-actin (1:2,000; Cell Signaling Technology, Danvers, United States), cleaved caspase-3 (1:1,000; Cell Signaling Technology), cleaved caspase-9 (1:1,000, Cell Signaling Technology), Bax (1:1,000; Cell Signaling Technology), multidrug resistance-associated protein 1 (MRP1; 1:1,000; Cell Signaling Technology), multi-drug resistance-1 (MDR1; 1:1,500; Cell Signaling Technology), phosphorylated (phospho)-PI3K (1:1,000; Cell Signaling Technology), PI3K (1:1,000; Cell Signaling Technology), phospho-AKT (1:1,000; Cell Signaling Technology), AKT (1:1,000; Cell Signaling Technology), phospho-mTOR (1:1,000; Cell Signaling Technology) and mTOR (1:1,000; Cell Signaling Technology). The protein levels were normalized to β-actin.

### *In vivo* Tumor Growth of SGC-7901/DDP Cells

The BALB/c-nu mice (5 weeks old; body weight: 15–19 g) were purchased from Guangdong Laboratory Experimental Animal Center (Guangzhou, China). The animals were housed in individual ventilated cage at 25.4 ± 2.2°C with 50.6 ± 8.8% humidity under controlled lighting (12 h light/day). All the animal experimental procedures were under the approval of Animal Ethic Committee of Nanjing Medical University. For the tumor inoculation and drug treatments, SGC-7901/DDP cells (1×10^7^ cells) were subcutaneously injected into the left flank of the nude mice. For the drug treatments, the mice from the vehicle group received phosphate buffered saline (2 ml/kg/day, i.p.); the mice from the DDP group received intraperitoneal DDP injection at 3 mg/kg/day; the mice from berberine group were treated with berberine (10 mg/kg/day); the mice from DDP + berberine group were injected with both DDP (3 mg/kg/day, i.p.) and berberine (10 mg/kg/day, i.p.). The tumor volume was measured every 5 days for 30 days. The formula for calculating tumor volume was as follow: volume = length × width × width/2. All treatments for 30 days, the animals were sacrificed by pentobarbitone (80 mg/kg, i.p.). The tumors were dissected and the weight of the tumors were weighed using a balance. The tumor tissues were then fixed for Ki-67 immunostaining and TUNEL assay.

### Ki-67 Immunostaining and TUNEL Assay

The proliferative potential of the tumor cells assessed by Ki-67 immunostaining. The Ki-67 immunostaining for the tumor tissues was performed according to previously published method (He et al., 2019). Briefly, the 4% paraformaldehyde-fixed tumor tissues were embedded in paraffin and sectioned into 4 µm thickness slices, and the slices were stained with Ki-67 (Cell Signaling Technology). The Ki-67-positive cells were analyzed using a confocal microscope by randomly choosing five fields. For the TUNEL assay, the sectioned tumor tissues (4 µm in thickness) were stained with TUENL *In Situ* Apoptosis Detection kit (Roche Diagnostic, Mannheim, Germany) according to previous studies (Ma et al., 2020). The TUNEL-positive cells were analyzed using a confocal microscope by randomly choosing five fields.

### Statistical Analysis

The statistical analyses were performed using GraphPad Prism Software (version 6.0; GraphPad Software, La Jolla, United States). Depending on the experiment type, two-tailed Student’s t-test or one-way ANOVA followed by Bonferroni’s multiple comparison tests was used for the analysis. The statistical significance was evaluated based on *p* values, and *p* < 0.05 was considered to indicate statistical significance.

## Results

### Effects of cisplatin and berberine on the cell viability gastric cancer cells and DDP-resistant gastric cancer cells

**FIGURE 1:**
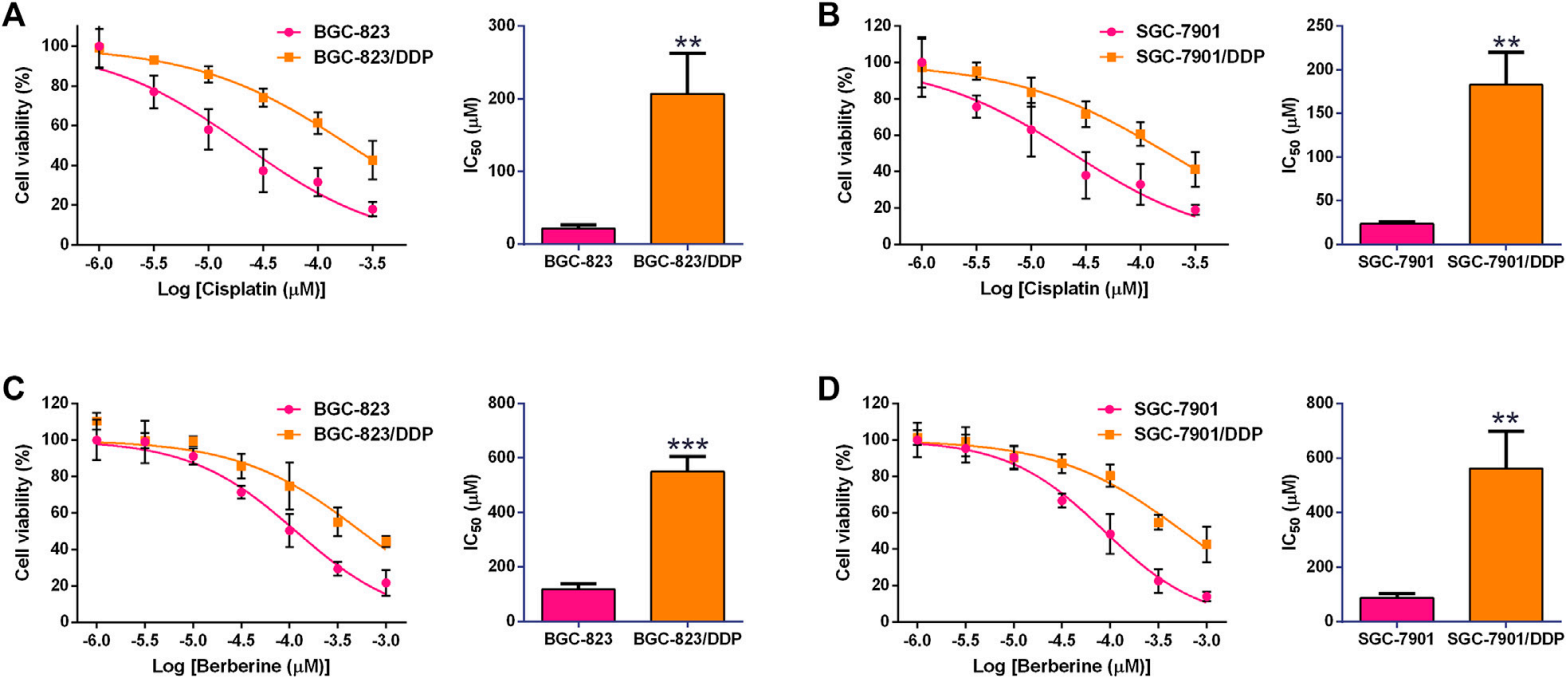
Effects of cisplatin and berberine on the cell viability gastric cancer cells and DDP-resistant gastric cancer cells. MTT assay determined the cell viability of BGC-823 and BGC-823/DDP cells **(A)**, and SGC-7901 and SGC-7901/DDP cells **(B)** after being treated with different concentrations of cisplatin. MTT assay determined the cell viability of BGC-823 and BGC-823/DDP cells **(C)**, and SGC-7901 and SGC-7901/DDP cells **(D)** after being treated with different concentrations of cisplatin. **p < 0.05 and ***p < 0.001 compared to the parental cell group (*n* = 3).

Firstly, we performed the MTT assay to examine the effects of DDP on the cell viability of the gastric cancer cells and DDP-resistant cells. In the BGC-823 and BGC-823/DDP cells, DDP dose-dependently inhibited the cell viability, and the IC_50_ of DDP in BGC-823/DDP cells was significantly higher than that in BGC-823 cells (BGC-823: 21.37 ± 5.13 µM vs. BGC-823/DDP: 206.8 ± 55.98 µM; Figure 1A). Similarly, DDP reduced the cell viability of SGC-7901 and SGC-7901/DDP cells in a concentration-dependent manner with the IC_50_ of DDP in the SGC-7901/DDP cells being remarkably higher than that in SGC-7901 cells (SGC-7901: 23.66 ± 2.14 µM vs. SGC-7901/DDP: 182.9 ± 32.71 µM; Figures 1B). These results suggest that BGC-823/DDP and SGC-7901/DDP exhibited resistance to the DDP treatment. Furthermore, we explored the effects of berberine on the cell viability of BGC-823 and BGC-823/DDP cells, and berberine at 30 µM started to exhibit inhibitory effects on the cell viability of BGC-823 and BGC-823/DDP cells, and berberine concentration-dependently supressed the cell viability of these cells. The IC_50_ of berberine in BGC-823/DDP cells was significantly higher than that in BGC-823 cells (BGC-823: 117.9 ± 20.49 µM vs. BGC-823/DDP: 549.6 ± 56.88 µM; Figures 1C). Consistent findings were observed in the SGC-7901 and SGC-7901/DDP cell lines (SGC-7901: 87.90 ± 15.23 µM vs. SGC-7901/DDP: 562.1 ± 135.9 µM; Figures 1B).

### Berberine Sensitizes DDP-Resistance Gastric Cancer Cells to Cisplatin Treatment

**FIGURE 2:**
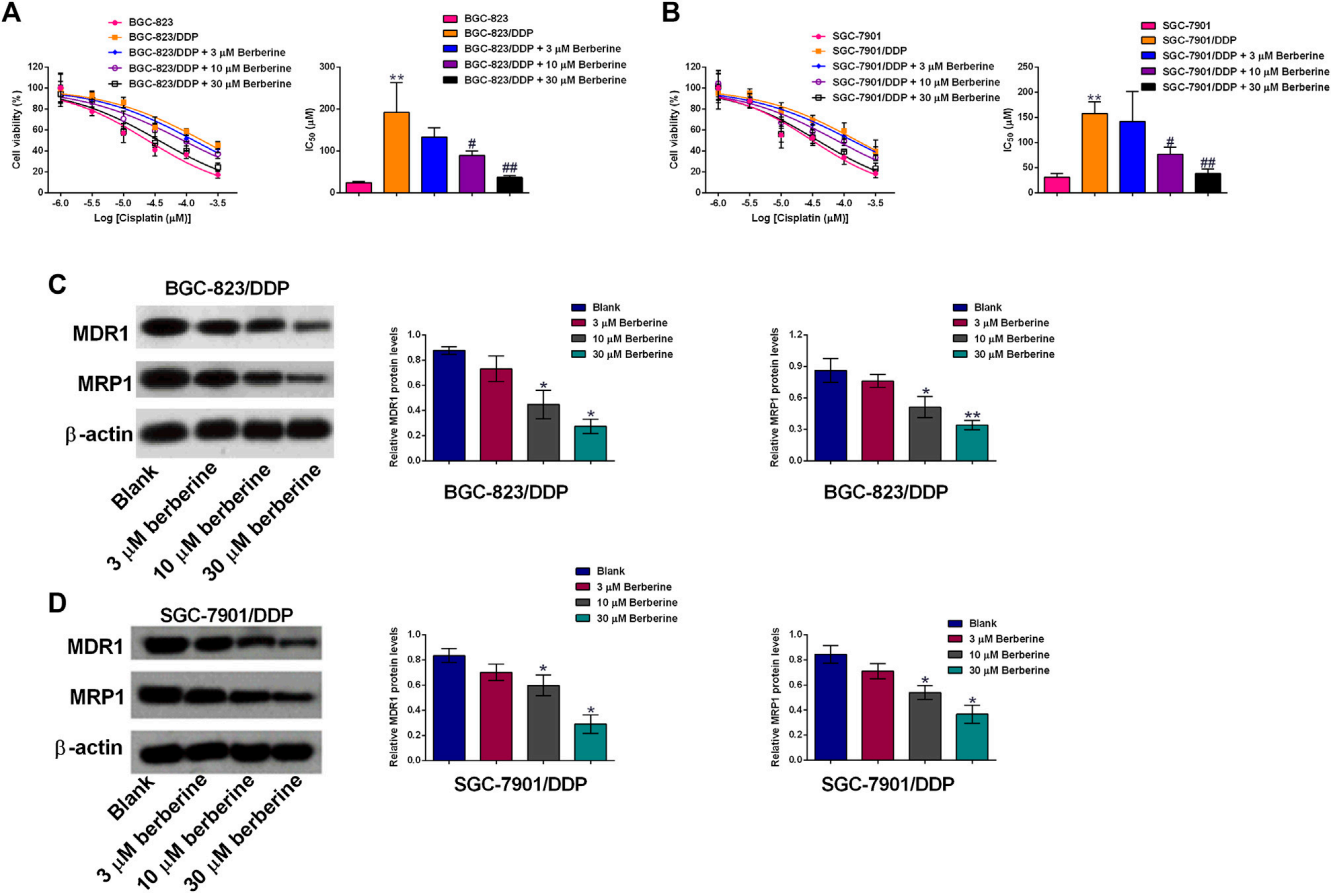
Berberine sensitizes DDP-resistance gastric cancer cells to cisplatin treatment. **(A)** MTT assay determined the cell viability of BGC-823 cells after cisplatin treatment or BGC-823/DDP cells after cisplatin or cisplatin + berberine treatments. **(B)** MTT assay determined the cell viability of SGC-7901 cells after cisplatin treatment or SGC-7901/DDP cells after cisplatin or cisplatin + berberine treatments. *N* = 3; **p < 0.01 compared to parental cell group; ^#^ *p* < 0.05 and ^##^ *p* < 0.01 compared to SGC-7901/DDP cells treated with DDP alone. **(C,D)** Western blot analysis of MDR1 and MRP1 protein levels in BGC-823/DDP and SGC-7901 cells after different concentrations of berberine treatments. *p < 0.05 and **p < 0.01 compared to Blank group (*n* = 3).

In order to examine if berberine could sensitize DDP-resistant gastric cancer cells to DDP, we co-treated BGC-823/DDP and SGC-7901/DDP cells with different concentrations of DDP and berberine. As shown in Figure 2A, 3 µM berberine treatment failed to significantly affect the IC_50_ values of DDP in BGC-823/DDP cells; whereas berberine at 10 and 30 µM significantly reduced the IC_50_ values of DDP in BGC-823/DDP cells when compared to BGC-823/DDP cells treated with DDP alone. Consistently, berberine at 10 and 30 µM significantly, but not 3 µM remarkably reduced the IC_50_ values of DDP in SGC-7901/DDP cells when compared the cells treated with DDP alone (Figure 2B). To gain into the mechanistic actions of berberine on the DDP-resistance, the protein levels of MDR1 and MRP1 were determined in both BGC-823/DDP and SGC-7901/DDP cells using Western blot analysis. DDP at 30 µM and berberine at 30 µM both caused a significant reduction of MDR1 and MRP1 protein levels in BGC-823/DDP cells when compared to Blank control group (Figure 2C). Importantly, berberine concentration-dependently down-regulated MDR1 and MRP1 protein expression when compared to non-treated BGC-823/DDP cells (Figure 2C). Consistent results were also shown in the SGC-7901/DDP cells (Figure 2D). These data indicated that berberine could sensitize BGC-823/DDP and SGC-7901/DDP cells to DDP possibly via down-regulating MDR1 and MRP1 protein expression.

### Berberine Promoted Cell Apoptosis of DDP-Resistant Gastric Cells With DDP Treatment

**FIGURE 3:**
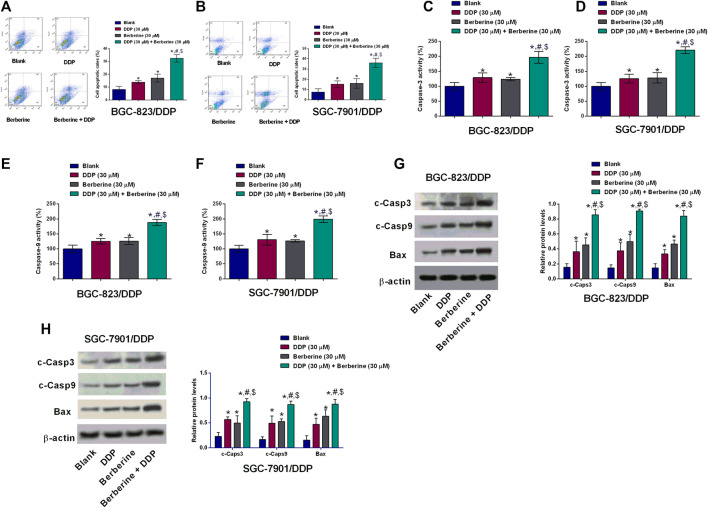
Berberine promoted cell apoptosis of DDP-resistant gastric cells with DDP treatment. **(A,B)** Flow cytometry of apoptotic BGC-823/DDP and SGC-7901/DDP cells after treatment with DDP (30 µM), Berberine (30 µM) or DDP (30 µM) + Berberine (30 µM). **(C,D)** Caspase-3 activity kit determined caspase-3 activity of BGC-823/DDP and SGC-7901/DDP cells after treatment with DDP (30 µM), Berberine (30 µM) or DDP (30 µM) + Berberine (30 µM). **(E,F)** Caspase-9 activity kit determined caspase-9 activity of BGC-823/DDP and SGC-7901/DDP cells after treatment with DDP (30 µM), Berberine (30 µM) or DDP (30 µM) + Berberine (30 µM). **(G,H)** Western blot determined cleaved caspase-3 (c-Caps3), cleaved caspase-9 (c-Caps9) and Bax protein levels in BGC-823/DDP and SGC-7901/DDP cells after treatment with DDP (30 µM), Berberine (30 µM) or DDP (30 µM) + Berberine (30 µM). *p < 0.05 compared to Blank group; ^#^ *p* < 0.05 compared to DDP (30 µM) group; ^$^ *p* < 0.05 compared to berberine (30 µM) group (*n* = 3).

The cell apoptosis of BGC-823/DDP and SGC-7901/DDP cells was evaluated by several experimental assays including flow cytometry, caspase-3 and -9 activities and western blot. DDP at 30 µM and berberine at 30 µM both slightly increased the cell apoptotic rates of BGC-823/DDP and SGC-7901/DDP cells when compared to Blank group (Figures 3A,B). Moreover, DDP and berberine co-treatment dramatically increased the BGC-823/DDP and SGC-7901/DDP cell apoptotic rates when compared to the other three groups (Figures 3A,B). Further examination of the capase-3 and -9 activities, we found that DDP and berberine co-treatment increased the caspase-3 and -9 activities by around two fold in BGC-823/DDP and SGC-7901/DDP cells; whereas DDP alone and berberine alone only caused a slightly increase in the capsase-3 and -9 activities of BGC-823/DDP and SGC-7901/DDP cells (Figures 3C–F). Moreover, the western blot analysis showed that the cleaved caspase-3,-9 and Bax protein levels were slightly increased after DDP or berberine treatment in both BGC-823/DDP and SGC-7901/DDP cells when compared to blank group (Figures 3G,H). Moreover, DDP and berberine co-treatment dramatically enhanced the protein expression of cleaved caspase-3,-9 and Bax in both BGC-823/DDP and SGC-7901/DDP cells when compared to the other three groups (Figures 3G,H).

### Berberine Sensitizes DDP-Resistance Gastric Cancer Cells to Cisplatin Treatment *In Vivo*

**FIGURE 4:**
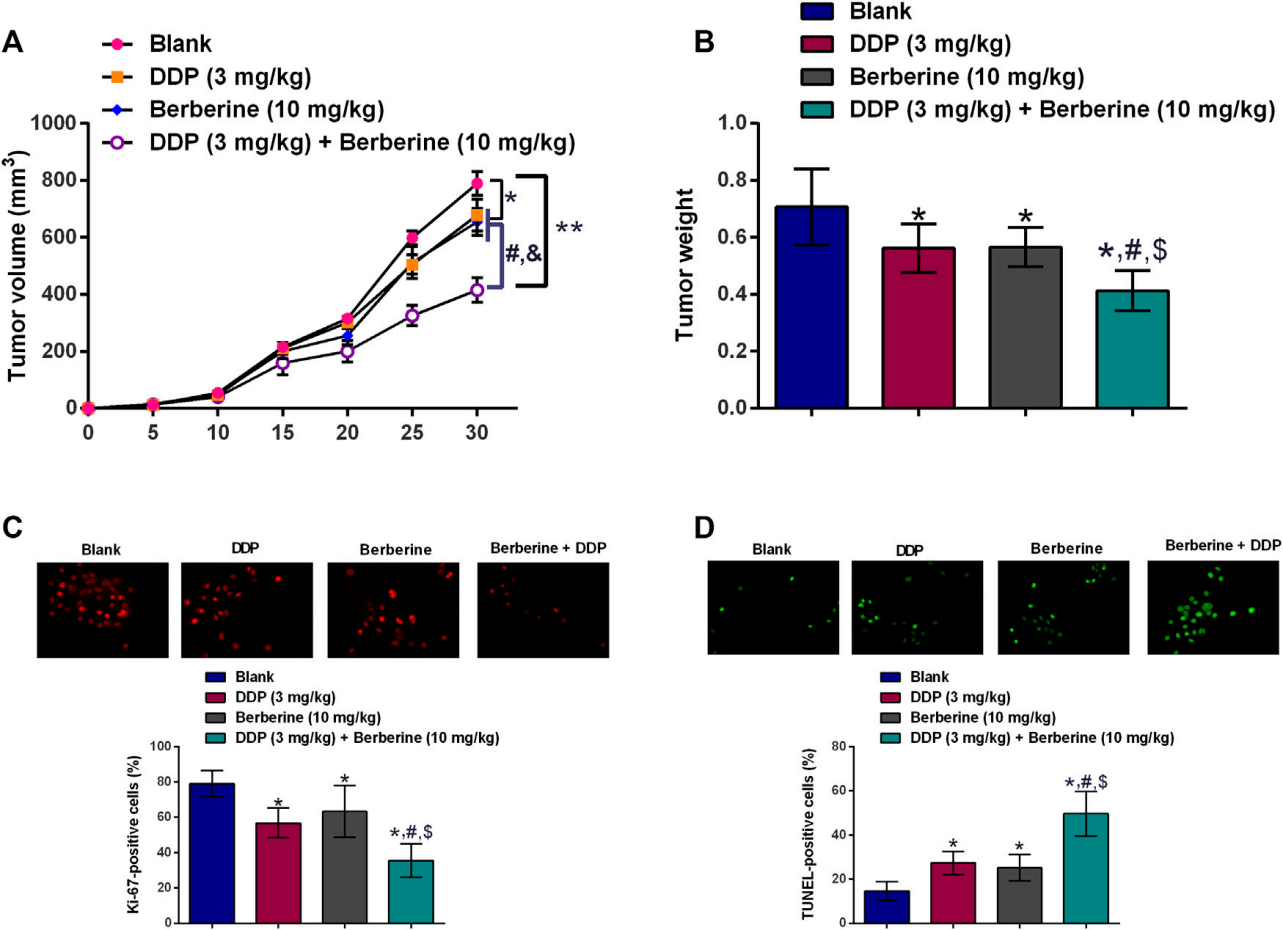
Berberine sensitizes DDP-resistance gastric cancer cells to cisplatin treatment *in vivo*. **(A)** *In vivo* tumor growth of SGC-7901/DDP cells in the nude mice after treatment with DDP (3 mg/kg, i.p.), berberine (10 mg/kg, i.p.) or DDP (3 mg/kg, i.p.) + Berberine (10 mg/kg, i.p.). **(B)** Tumor weight dissected from the nude mice after treatment with DDP (3 mg/kg, i.p.), berberine (10 mg/kg, i.p.) or DDP (3 mg/kg, i.p.) + Berberine (10 mg/kg, i.p.). **(C)** Immunostaining of Ki-67 in the tumor tissues from the mice after treatment with DDP (3 mg/kg, i.p.), berberine (10 mg/kg, i.p.) or DDP (3 mg/kg, i.p.) + Berberine (10 mg/kg, i.p.). **(D)** TUNEL assay determined cell apoptosis in the tumor tissues from the mice after treatment with DDP (3 mg/kg, i.p.), berberine (10 mg/kg, i.p.) or DDP (3 mg/kg, i.p.) + Berberine (10 mg/kg, i.p.). *p < 0.05 compared to Blank group; ^#^ *p* < 0.05 compared to DDP (3 mg/kg) group; ^$^ *p* < 0.05 compared to berberine (10 mg/kg) group (*n* = 6).

The *in vivo* growth of SGC-7901/DDP cells were evaluated in a xenograft mice mode, DDP (3 mg/mg/day) or berberine (10 mg/kg/day) treatment caused a trivial suppression in the tumor growth of SGC-7901/DDP cells when compared to blank group (Figure 4A). Moreover, co-treatment with DDP and berberine suppressed the *in vivo* tumor growth of SGC-7901/DDP cells by around 50% when compared to blank group (Figure 4A). The examination of tumor weight showed the consistent results (Figure 4B). Further the proliferative potential and apoptosis of tumor tissues were assessed by Ki-67 immunostaining and TUENL assay. As shown in Figures 4C,D, DDP or berberine alone slightly suppressed the number of Ki-67-positive cells and increased the number of TUNEL-positive cells, when compared to blank group. Moreover, a dramatic reduction in the number of Ki-67-positive cells and an increase in the number of TUNEL-positive cells were observed in the tumor tissues from DDP and berberine co-treatment group (Figures 4C,D).

### Berberine inhibited PI3K/AKT/mTOR signaling in the DDP-resistant gastric cancer cells with cisplatin treatment

**FIGURE 5:**
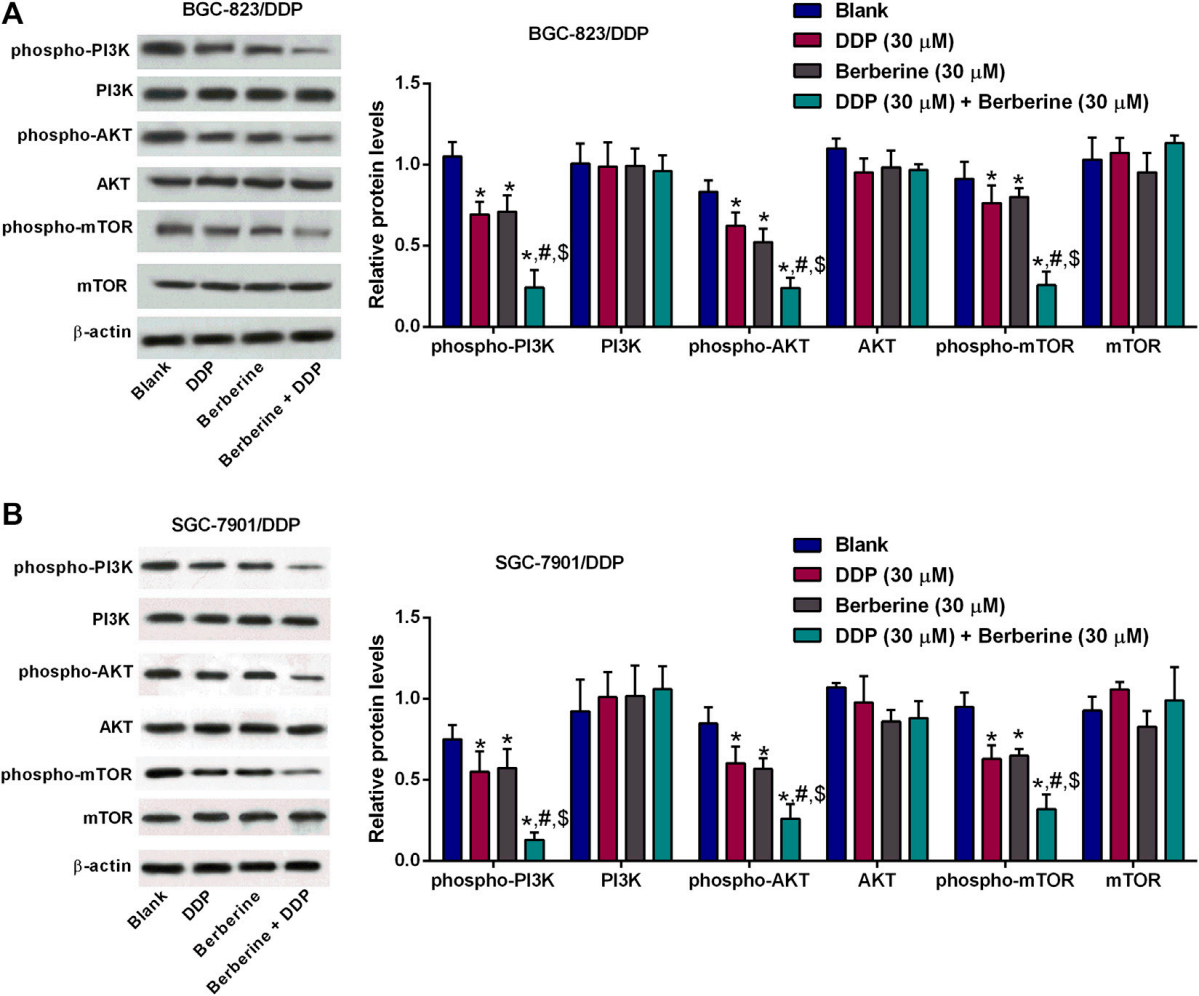
Berberine inhibited PI3K/AKT/mTOR signaling in the DDP-resistant gastric cancer cells with cisplatin treatment. **(A,B)** Western blot determined phosphor-PI3K, PI3K, phospho-AKT, AKT, phosphor-mTOR or mTOR protein levels in BGC-823/DDP and SGC-7901/DDP cells after treatment with DDP (30 µM), Berberine (30 µM) or DDP (30 µM) + Berberine (30 µM). *p < 0.05 compared to Blank group; ^#^ *p* < 0.05 compared to DDP (30 µM) group; ^$^ *p* < 0.05 compared to berberine (30 µM) group (*n* = 3).

The effects of berberine on the PI3K/AKT/mTOR signaling were further examined by western blot assay. The phospho-PI3K,-AKT and -mTOR protein levels were slightly reduced after DDP or berberine treatment in both BGC-823/DDP and SGC-7901/DDP cells when compared to blank group (Figures 3G,H). Moreover, DDP and berberine co-treatment dramatically enhanced the protein expression of phospho-PI3K, -AKT and -mTOR in both BGC-823/DDP and SGC-7901/DDP cells when compared to the other three groups (Figures 5A,B).

## Discussion

DDP has been widely used as the chemotherapeutic drugs for treating different types of human malignancies; however, the intrinsic and acquired DDP-resistance during the course of the chemotherapy largely hindered the clinical use of DDP in these patients (Venerito et al., 2018). On the other hand, the severe side effects of DDP are also an obstacle during the clinical application. Thus, identifying novel targets/strategies to promote the sensitivity of gastric cancer cells to DDP is of great importance. In this study, we established the DDP-resistant gastric cancer cells, where the IC_50_ values of DDP in the BGC-823/DDP and SGC-7901/DDP were significantly higher than that in the corresponding parental cells. Berberine could concentration-dependently inhibited the cell viability of BGC-8203 and SGC-7901 cells; while the inhibitory effects of berberine on the cell viability were largely attenuated in the DDP-resistant cells. Berberine pre-treatment significantly sensitized BGC-823/DDP and SGC-7901/DDP cells to DDP. Furthermore, berberine treatment concentration-dependently down-regulated the MRP1 and MDR1 protein levels in the BGC-823/DDP and SGC7901/DDP cells. Interestingly, the cell apoptosis of BGC-823/DDP and SGC-7901/DDP cells was significantly enhanced by co-treatment with berberine and DDP. The results from animals also showed that berberine treatment sensitized SGC-7901/DDP cells to DDP *in vivo*. Mechanistically, berberine significantly suppressed the PI3K/AKT/mTOR in the BGC-823/DDP and SGC-7901/DDP cells treated with DDP. Taken together, our results indicated that berberine sensitized DDP-resistant gastric cancer cells to DDP via enhanced cell apoptosis and inhibited PI3K/AKT/mTOR signaling.

The anti-tumor effects of berberine in gastric cancer have been illustrated in various studies. Berberine was effective to induce cell cycle arrest and apoptosis in human gastric carcinoma SNU-5 cells (Lin et al., 2006), and berberine-induced gastric cancer cell apoptosis is associated with Akt signaling (Yi et al., 2015). Moreover, berberine inhibited SNU-5 cell migration via down-regulating metalloproteinase-1, -2 and -9 expression (Lin et al., 2008). Wang et al., showed that berberine enhanced the anti-tumor effects of EGFR inhibitors in gastric cancer via supressing EGFR signaling (Wang et al., 2016). A recent study by Hu et al., showed that berberine attenuated gastric carcinoma proliferation, invasion, and migration by targeting the AMPK/HNF4α/WNT5A signaling (Hu et al., 2018). In agreement with previous findings, we also demonstrated the berberine exerted tumor-suppressive effects on the gastric carcinoma cell lines in a concentration-dependent manner. In the gastric cancer cells with drug-resistance, berberine could target surviving and STATs to sensitize gastric cancer cells to 5-Fluorouracil (Pandey et al., 2015). Consistently, our data showed that berberine sensitizes BGC-823/DDP and SGC-7901/DDP cells to DDP in a concentration-dependent manner. MDR1 is encoded by the ABCB1 gene with a molecular weight of 170 kDa, and studies found that MDR1 is highly expressed in tissues of the gastric carcinoma, and up-regulation of MDR1 was closed correlated with chemo-resistance of the gastric cancer cells (Lage, 2003). MDR1 exerted its effects via transporting toxic substances and intracellular drugs to extracellular space. MRP1 is encoded by the ABCC1 gene and belong to the superfamily of ATP-binding cassette transporters. MRP1 was also found to be up-regulated in the DDP-resistant gastric cancer cells, and MRP1 enhanced chemo-resistance via transporting chemotherapeutic drugs to extracellular space (Lage, 2003). To uncover the relationship of MDR1 and MRP1 with berberine-mediated DDP-sensitivity, we further examined the effects of berberine on the MDR1 and MRP1 protein expression. The expression of MDR1 and MRP1 proteins was significantly down-regulated by berberine in the BGC-823/DDP and SGC-7901/DDP cells. These results implied that MDR1/MRP1 pathway could participate in the berberine-mediated DDP-sensitivity in gastric cancer.

The effects of berberine on the cell apoptosis have been well-documented in various types of cancers. Berberine in combination with DDP induces apoptosis in ovarian cancer cells (Liu et al., 2019). Berberine enhances chemosensitivity by inducing apoptosis via dose-orchestrated AMPK signaling in breast cancer (Pan et al., 2017). More importantly, berberine promoted gastric cancer cell apoptosis via regulating Akt signaling (Yi et al., 2015). Further studies showed hat berberine-induced apoptosis was associated with caspase activation (Wang et al., 2020). In the study, we consistently showed that berberine plus DDP significantly induced apoptosis in BGC-823/DDP and SGC-7901/DDP cells. In addition, the caspase-3 and -9 activities and protein levels were significantly increased in these cells upon berberine plus DDP treatment. These results implied that berberine-mediated DDP-sensitivity in gastric cancer cells may be associated with enhanced apoptosis by caspase-3 and -9 activation.

PI3K/Akt/mTOR signaling axis has been regarded as an important pathway in regulating drug-resistance in different cancer types including gastric cancer. Studies have demonstrated that phosphorylation of Akt and mTOR was increased in DDP-resistant gastric cancer cells and inhibition of PI3K/Akt pathway significantly attenuated CCL2-mediated DDP-resistance in gastric cancer (Xu et al., 2018). Dual inhibitor of PI3K and mTOR (NVP-BEZ235) promoted the drug resistance of gastric cancer cells to 5-fluorouracil (Li et al., 2018). TSPAN9 enhance the resistance of gastric cancer to 5-fluorouracil by activating PI3K/AKT/mTOR-mediated autophagy (Qi et al., 2020). On other hand, berberine could ameliorate diabetes-associated cognitive decline and hepatic ischemia/reperfusion injury via down-regulating PI3K/Akt/mTOR signaling pathway (Sheng et al., 2015; Chen et al., 2017). Berberine in combination with solid lipd curcumin particles could also increase cell death by inhibiting PI3K/Atk/mTOR pathway in glioblastoma cells (Maiti et al., 2019). In our results, the phosphorylation of PI3K, AKT and mTOR was remarkably repressed by the treatment with berberine plus DDP in BGC-823/DDP and SGC-7901/DDP cells. This indicated that berberine-mediated DDP-sensitivity of gastric cancer cells might be associated with inhibition of PI3K/AKT/mTOR signaling.

Our current findings are still at the preliminary stages, and further molecular mechanisms of berberine-mediated effects on the chemo-sensitivity should be investigated. Our results showed that berberine improved the DDP-sensitivity of gastric cancer cells, and berberine enhanced the chemo-sensitivity possibly via attenuating the expression of chemo-resistance associated mediators, which still require further examination. Recent studies also demonstrated that berberine exerted anti-tumor effects via regulating the expression of non-coding RNAs (Wei et al., 2016; Chang, 2017; Wang and Zhang, 2018), and further studies may use the non-coding RNA microarrays to determine the berberine-mediated downstream non-coding RNAs associated with chemosensitivity of the gastric cancer. The present study has not systematically examined the toxic effects of berberine, which may be considered in the future studies. Besides, berberine has also been reported to regulate the angiogenesis in the tumor tissues (Jin et al., 2018; Luo et al., 2019; Lopes et al., 2020), whether berberine-mediated angiogenesis-associated mechanisms contributes to the chemo-sensitivity of the gastric cancer cells should be further explored.

In conclusion, we observed that berberine sensitizes gastric cancer cells to DDP. Further mechanistic findings suggested that berberine-mediated DDP-sensitivity may be associated with reduced expression of drug transporters (MDR1 and MRP1), enhanced apoptosis and repressed PI3K/AKT/mTOR signaling.

## Data Availability Statement

The original contributions presented in the study are included in the article/Supplementary Material, further inquiries can be directed to the corresponding authors.

## Ethics Statement

The animal study was reviewed and approved by Animal Ethic Committee of Nanjing Medical University.

## Author Contributions

WW and MZ conceived and designed the study; YK and BT performed the experiments; BT and WW contributed to the data analysis and the critical reading of manuscript. WW and MZ wrote the manuscript. All authors read and approved the final manuscript

## FUNDING

This work was supported by the Fund of Administration of Traditional Chinese Medicine of Jiangsu Province of China (YB201987).

## Conflict of Interest

The authors declare that the research was conducted in the absence of any commercial or financial relationships that could be construed as a potential conflict of interest.
