# Supplementary material for: Berberine Improves Chemo-Sensitivity to Cisplatin by Enhancing Cell Apoptosis and Repressing PI3K/AKT/mTOR Signaling Pathway in Gastric Cancer
Source: Front Pharmacol. 2020 Dec 9;11:616251. doi: 10.3389/fphar.2020.616251 (PMC7756080; doi:10.3389/fphar.2020.616251)

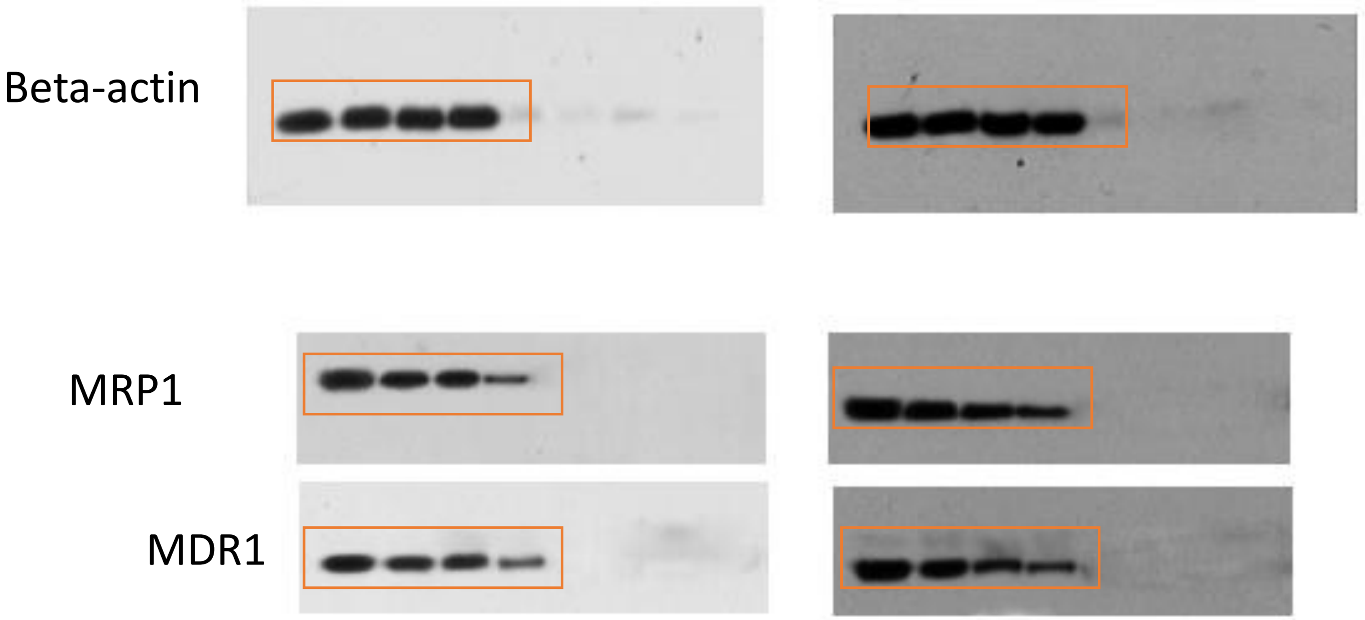

Figure 2C Figure 2D

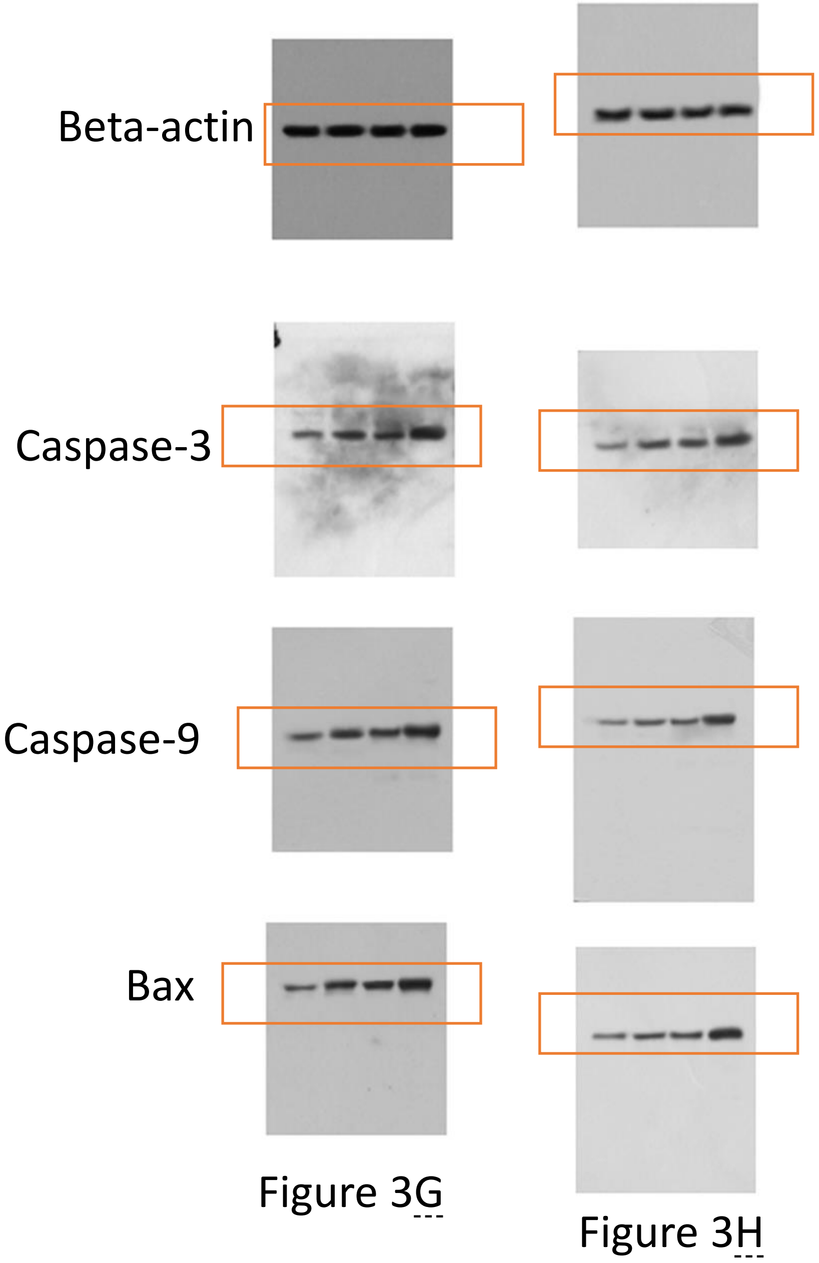
Beta-actin
Caspase-3
Caspase-9
Bax
Figure 3G
Figure 3H

Figure 5A

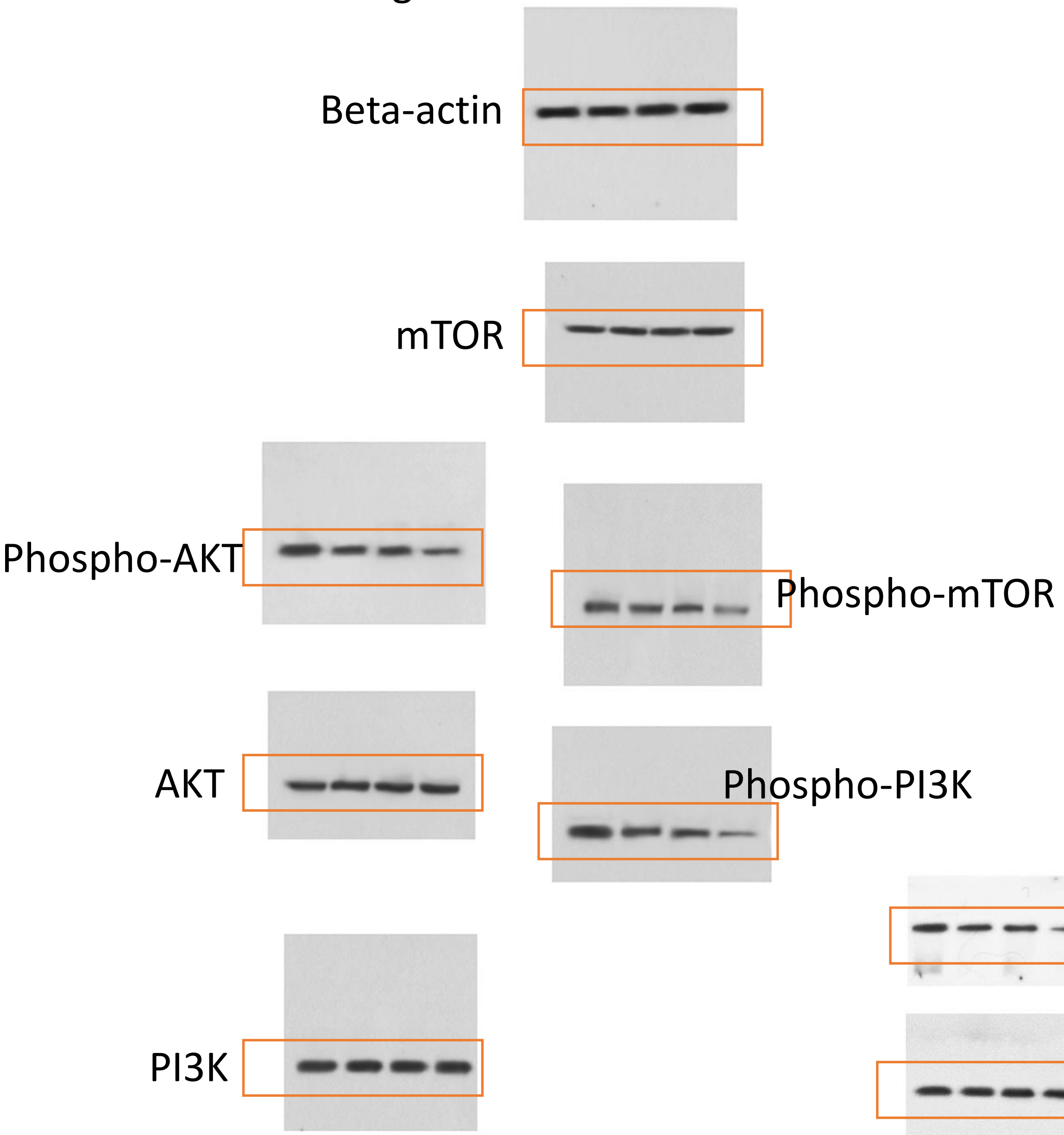

Figure 5B

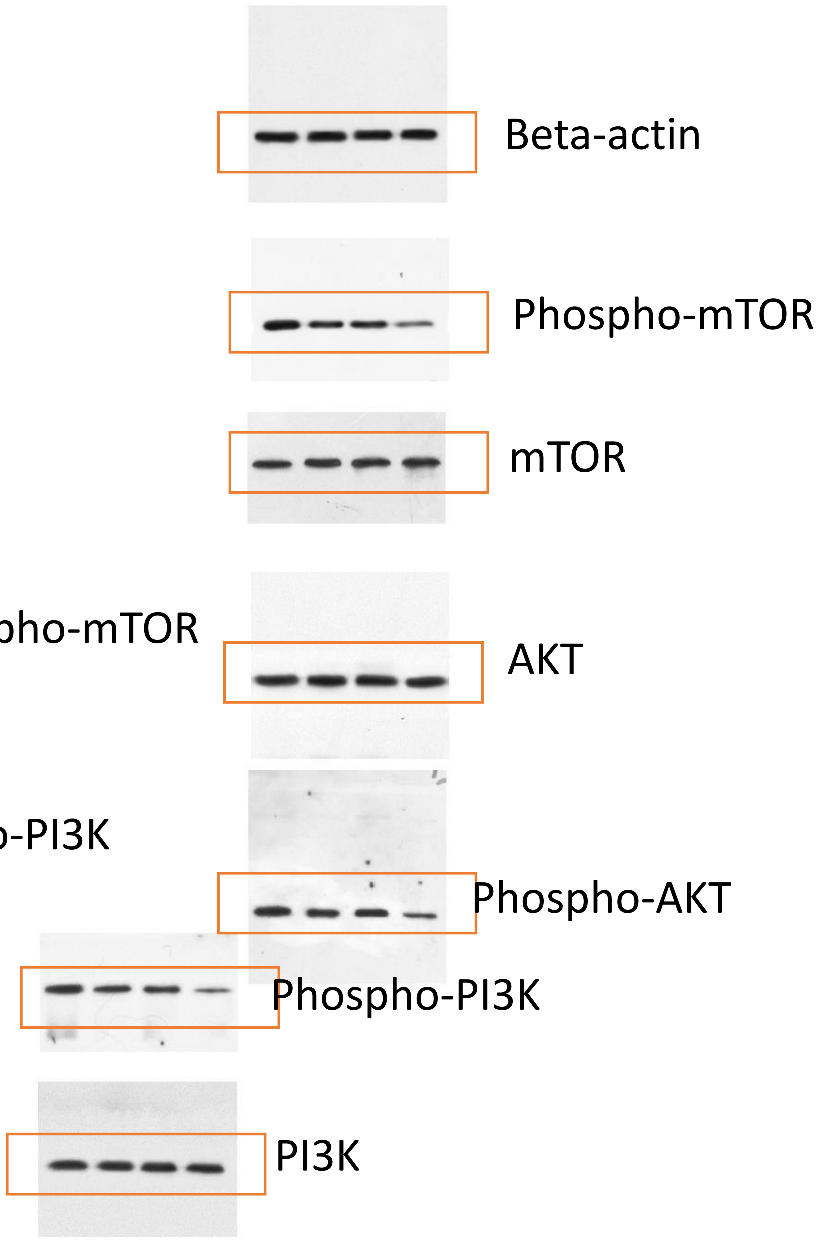

Supplement: Supplementary file 1 [file datasheet1.pdf]
