# Supplementary material for: Berberine Improves Chemo-Sensitivity to Cisplatin by Enhancing Cell Apoptosis and Repressing PI3K/AKT/mTOR Signaling Pathway in Gastric Cancer
Source: Front Pharmacol. 2020 Dec 9;11:616251. doi: 10.3389/fphar.2020.616251 (PMC7756080; doi:10.3389/fphar.2020.616251)
Supplement: Supplementary file 2 [file datasheet1.zip › Figure legends.docx]

**Supplemental figure legends**

Figure S1. Beta-actin expression for Figure 2C.

Figure S2. MRP1 expression for Figure 2C.

Figure S3. MDR1 expression for Figure 2C.

Figure S5. Beta-actin expression for Figure 2D.

Figure S5. MRP1 expression for Figure 2D.

Figure S6. MDR1 expression for Figure 2D.

Figure S7. Beta-actin expression for Figure 3G.

Figure S8. Caspase-3 expression for Figure 3G.

Figure S9. Caspase-9 expression for Figure 3G.

Figure S10. Bax expression for Figure 3G.

Figure S11. Beta-actin expression for Figure 3H.

Figure S12. Caspase-3 expression for Figure 3H.

Figure S13. Caspase-9 expression for Figure 3H.

Figure S14. Bax expression for Figure 3H.

Figure S15. Phosphor-PI3K expression for Figure 5A.

Figure S16. PI3K expression for Figure 5A.

Figure S17. Phosphor-AKT expression for Figure 5A.

Figure S18. AKT expression for Figure 5A.

Figure S19. Phosphor-mTOR expression for Figure 5A.

Figure S20. mTOR expression for Figure 5A.

Figure S21. Beta-actin expression for Figure 5A.

Figure S22. Phosphor-PI3K expression for Figure 5B.

Figure S23. PI3K expression for Figure 5B.

Figure S24. Phosphor-AKT expression for Figure 5B.

Figure S25. AKT expression for Figure 5B.

Figure S26. Phosphor-mTOR expression for Figure 5B.

Figure S27. mTOR expression for Figure 5B.

Figure S28. Beta-actin expression for Figure 5B.
