# Supplementary figures and images for: Berberine Improves Chemo-Sensitivity to Cisplatin by Enhancing Cell Apoptosis and Repressing PI3K/AKT/mTOR Signaling Pathway in Gastric Cancer
Source: Front Pharmacol. 2020 Dec 9;11:616251. doi: 10.3389/fphar.2020.616251 (PMC7756080; doi:10.3389/fphar.2020.616251)

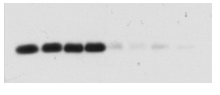

Supplement: Supplementary file 2 [file datasheet1.zip › Figure S1.tif]

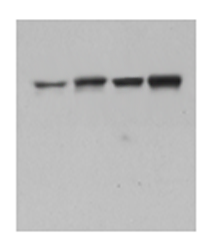

Supplement: Supplementary file 2 [file datasheet1.zip › Figure S10.tif]

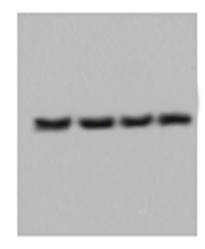

Supplement: Supplementary file 2 [file datasheet1.zip › Figure S11.tif]

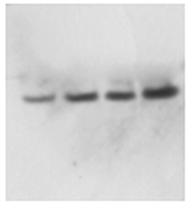

Supplement: Supplementary file 2 [file datasheet1.zip › Figure S12.tif]

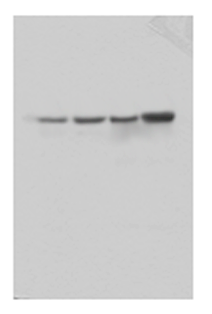

Supplement: Supplementary file 2 [file datasheet1.zip › Figure S13.tif]

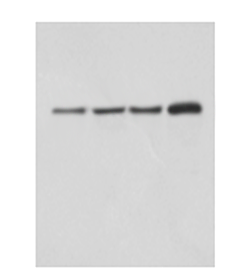

Supplement: Supplementary file 2 [file datasheet1.zip › Figure S14.tif]

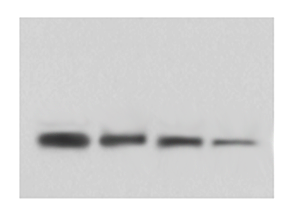

Supplement: Supplementary file 2 [file datasheet1.zip › Figure S15.tif]

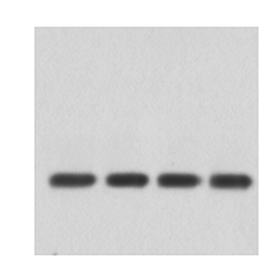

Supplement: Supplementary file 2 [file datasheet1.zip › Figure S16.tif]

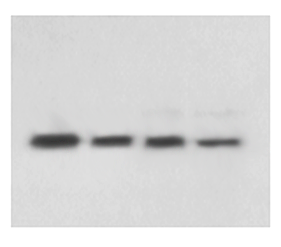

Supplement: Supplementary file 2 [file datasheet1.zip › Figure S17.tif]

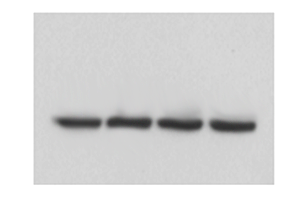

Supplement: Supplementary file 2 [file datasheet1.zip › Figure S18.tif]

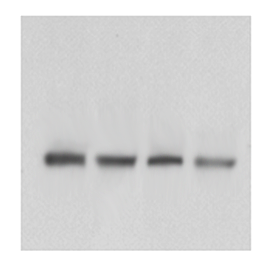

Supplement: Supplementary file 2 [file datasheet1.zip › Figure S19.tif]

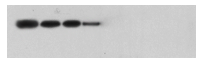

Supplement: Supplementary file 2 [file datasheet1.zip › Figure S2.tif]

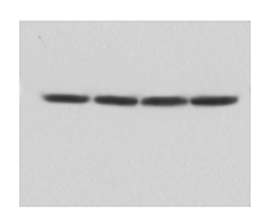

Supplement: Supplementary file 2 [file datasheet1.zip › Figure S20.tif]

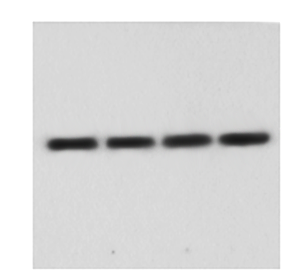

Supplement: Supplementary file 2 [file datasheet1.zip › Figure S21.tif]

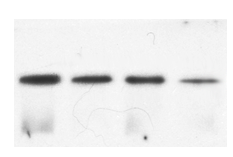

Supplement: Supplementary file 2 [file datasheet1.zip › Figure S22.tif]

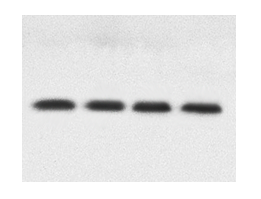

Supplement: Supplementary file 2 [file datasheet1.zip › Figure S23.tif]

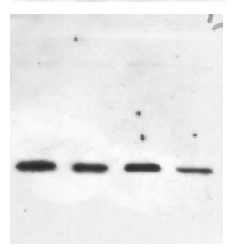

Supplement: Supplementary file 2 [file datasheet1.zip › Figure S24.tif]

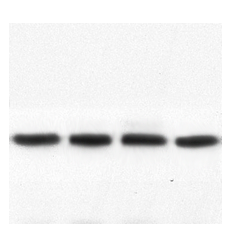

Supplement: Supplementary file 2 [file datasheet1.zip › Figure S25.tif]

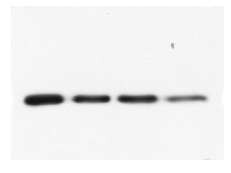

Supplement: Supplementary file 2 [file datasheet1.zip › Figure S26.tif]

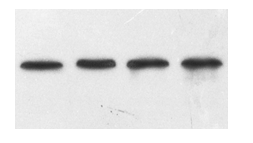

Supplement: Supplementary file 2 [file datasheet1.zip › Figure S27.tif]

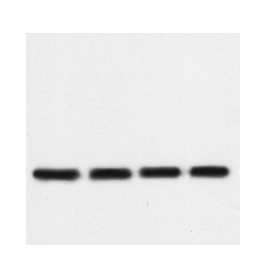

Supplement: Supplementary file 2 [file datasheet1.zip › Figure S28.tif]

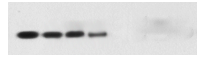

Supplement: Supplementary file 2 [file datasheet1.zip › Figure S3.tif]

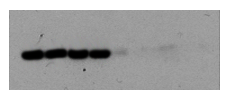

Supplement: Supplementary file 2 [file datasheet1.zip › Figure S4.tif]

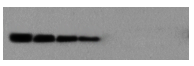

Supplement: Supplementary file 2 [file datasheet1.zip › Figure S5.tif]

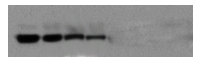

Supplement: Supplementary file 2 [file datasheet1.zip › Figure S6.tif]

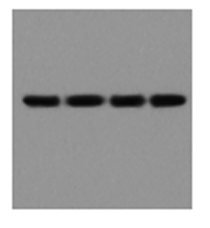

Supplement: Supplementary file 2 [file datasheet1.zip › Figure S7.tif]

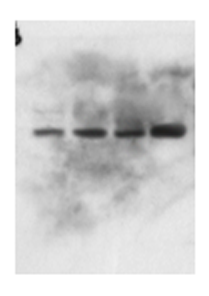

Supplement: Supplementary file 2 [file datasheet1.zip › Figure S8.tif]

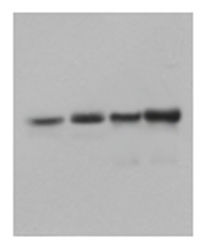

Supplement: Supplementary file 2 [file datasheet1.zip › Figure S9.tif]
